# Preservation of the Uterus in Removal of Chronic Infected Adnexa

**Published:** 1919-04

**Authors:** F. C. Floeckinger

**Affiliations:** Taylor, Texas


### Preservation of the Uterus in Removal of Chronic Infected Adnexa.

DR. F. C. FLOECKINGER,
Taylor, Texas.

The question whether to preserve the uterus or remove same in the course of an operation for infected adnexa has been pretty clearly solved, through the painstaking work in the laboratory, as well as in the operating room and at the bedside, of men like John A. Sampson of New York, Goetsch of Baltimore, Bell of Liverpool, Schickele of Germany, Polak of Brooklyn, Graves of Boston and several others, who have laid down the principles involved as to when and when not to preserve the uterus. In former days we were mostly guided simply by the gross pathological appearance of the structures during exploration. And up to this day we have surgeons who rely on the gross pathology and do not consider the functions, the relationship between the different reproducing organs. Therefore the end results of operations on the reproducing organs are primary and not always what we expect. We generally promise a patient a complete recovery after undergoing an operation, and when it comes down to the facts we must justly admit that results in many cases are far from being satisfactory to the patient as well as to the surgeon. It is of the greatest importance to know when to make a complete ablation or when to do conservative surgery.

We surgeons are all procrastinating. Today we adopt a principle, which we follow for a time, only to completely overthrow the same later. There was a time when ovaries were re-

*Paper read before the Central Texas Medical Association at Waco, Texas, 1918.

moved for almost every possible and impossible ailment of the body. When a complete removal of the uterus and ovaries after the menopause had been performed, naturally the ablation symptoms were missing, due to the retrograde function of the corpus luteum. But those cases in which the ovaries were removed during the childbearing period became complete physical as well as mental wrecks. Therefore understanding the unfortunate results after oophorectomy in the active childbearing period, the conservative period had its beginning. And up to this day the end results are far from being satisfactory to the surgeon and patient as well. I am not in favor of conservatism, but I like to know when to be radical and when to be conservative. Schickele and Walthard have given us statistics of end results, which are far from being satisfactory. Those cases in which the uterus was extirpated with the tubes and the ovaries left showed unsatisfactory results. Those men have made extensive experiments and have presented us with facts, which will be standards for the future.

We know that the corpus luteum embodies the principle of ovarian secretion. The corpus luteum is the organ of the internal secretion and its secretion controls the menstruation by sensitizing the endometrium. Several authors claim that the uterus produces a certain internal secretion, which in turn is neutralized by the internal secretion of the ovary. According to Graves, of Boston, there exists between the ovaries and the uterus a harmony in the internal secretion, which becomes disturbed if the function of one or the other is abolished. Most of us have had cases in which genital psychoneurosis was present in a rudimentary uterus and normally developed ovaries, when a complete removal of the uterus and ovaries brought a perfect recovery. Under normal conditions there is established a certain equilibrium between the different ductless glands in the female, and it is the disharmony, as I may call it, which brings forth the neurotic symptoms after conservative operations on the female genitaliae. Graves, of Boston, in his citation of Walthard, states that inasmuch as the normal function of the ovary is depended on its physiological connection with the uterus, a disturbance or severance of this connection supposedly produces an ovarian disfunction, which in turn is likely to upset the balance of the other ductless glands, thus creating in them a corresponding disfunction.

During the radical period, in which ovaries were removed ad libitum, the ablation symptoms were most marked in those cases in which the ovaries alone were removed and the uterus preserved. This in itself establishes the fact of the interrelationship of the uterus and the ovaries.

Statistics have shown us another factor worth while to consider. In the removal of the ovaries in the type of young woman, which we would classify as neurotic, the ablation symptoms were more marked. The results after total extirpation of the voaries in the childbearing period in those neurotic women brought forth a symptom complex, which could without any question be attributed to changes in the vasomotor system.

Inasmuch as the secretory reaction exerts itself principally on the symphathetic nervous system, the vasomotor symptom complex will exert itself mostly in those types of patients which we would classify under the neurotic type. There seems to be an individual variation in the power of adaptation to the changes in the secretory system.

After this preliminary recapitulation of the relationship between the uterus and ovaries, I come to the practical side of this paper. The question involved is this: What course should we adopt to give our patients the best results? We have all had cases in which after hysterectomy with conservation of the ovaries the end results were not satisfactory and we had to do a secondary ablation which was attended with splendid results. It is the interrelationship which has been disturbed and the equilibrium must be established between the ovarian function and the uterus to get good results.

I wish to state a case in which a woman of 36 suffered from severe menorrhagia, in which it was impossible to find the cause. In addition the patient would suffer from intense nervous irritability and vasomotor disturbances. Those disturbances I attributed mostly to the mental strain and irritability due to prolonged metrorrhagias and I removed the uterus, doing a vaginal hysterectomy. I found out to my regret that the vasomotor disturbances were not alleviated at all and the patient began to present symptoms of the severest type of hysteria. Those symptoms, as I found out, were not due to a genital neurosis, but were due to hyperfunction of the ovaries. A double oophorectomy was done and in a short time the patient made an uninterrupted recovery with the disappearance of all the nervous symptoms.

The macroscopical as well as microscopical examination of the ovaries did not show the slightest pathological condition. The ovaries also were larger than on an average woman. No doubt in this case we have a typical picture of disharmony.

In chronic infections of the adnexa we will find always an inflammation of the uterus present. This inflammatory condition of the body of the uterus is more marked in infection due to the mixed type, such as staphylo- or streptococcus or baccillus coli, than the infection due to the Neisserian bacillus. Polak, of Brooklyn, has made extensive investigation in this line and we can only concur with his statements. A mixed infection, as we find after abortion or puerperal infection, will produce a much more severe metritis than the Neisserian infection will. In an infection due to the mixed type we will find occasionally only one tube infected involving nearly always the ovary also. The uterus is much more enlarged than in the Neisserian type. This condition is due to the direct invasion of the subendometrial tissue and very often due to the use of the uterine curette, especially if same is used during the acute stage of infection. A Neisserian infection will travel on the surface of the uterine mucosa and will always involve both tubes. But experiments have shown that as soon as the Neisserian bacillus reaches the ostium uternum, it begins to involve deeper tissue of the Fallopian tube. To understand this is very easy. Just get a section of the fundus of the uterus, one from the cornua of the tube and another one from any part of the tube. The mucosa of the tube gives the bacteria a much better field for propagation, as same is considerably more developed than in the uterus. The result will be that the infection will spread to the surrounding tissue causing adhesions to the neighboring organs, such as the ovary, and causing a gradual destruction of the ovarian tissue.

The question which arises is this: If the ovaries are very little affected by inflammatory conditions, shall we remove the tubes or not? This depends entirely on two factors. First of all it depends on the extent of the inflammatory condition; second it depends on the blood supply of the ovaries. Polak claims that it is the bad company the ovary is in, and not the ovary primarily which causes the macroscopical pathological conditions. Very properly he advises that if the blood supply to the ovary is not interfered with, save the ovary. Inasmuch as the saving of the ovaries and the extirpation of the uterus would cause a disharmony in the internal secretion, it becomes necessary to do conservative surgery. In specific infections we find mostly two places in the uterus which are affected more than the others. Those two places are the cervix and the uterine end of the tube.

A simple ligation of the tube at the uterine end is bad surgery, inasmuch as it always leave a focus of infection behind and the patient will suffer from leucorrhoea and prolonged menstrual period. Therefore in all those cases in which the ovaries are preserved, the isthmus of the tube should be removed completely by extirpating a part of the uterine wall. It is immaterial which method a surgeon uses in removing a part of the uterus, as long as he does away with the infection focus and does not disturb the interrelationship between uterus and ovary.

Beuttner, of Geneva, Switzerland, and Bell, of Liverpool, have adopted this resection with preservation of the function of the endometrium for several years with splendid results. But, as stated before, it is immaterial what method you use, as long as you succeed in removing the focus of infection. In removal of the tubes with preservation of the ovaries great stress should be laid on the careful handling of the ovarian tissue. The surface epithelium of the ovary is very delicate, and if adhesions have been severed, you certainly can count on it that adhesions will form again.

In extensive inflammatory conditions of the tube we always will find also the ovaries adhering to the surrounding tissues, mostly the posterior wall of the uterus. The ovary will give you a picture of beginning cystic degeneration and if you save those ovaries you certainly will have trouble afterwards. Not a long time ago I had a case in which three years previously I had removed both tissues and the left ovary for a specific infection. The uterus was at that time saved. The patient never did get well, did not gain in strength, and became a neurasthenic. Fluor albus and prolonged menstruation were constant. A few months ago I opened the abdomen and found that the right ovary was cystic, degenerated, the size of a newborn baby's head, and the uterus very much infiltrated. I performed a supracervical hysterectomy and removed the remaining ovary and the woman, about 30 years of age, made a most perfect recovery without any manifestation of disharmony.

In former times I used to transplant such infected ovaries between the two folds of the broad ligament, only to have to remove same later. In all those cases it is proper if the uterus is completely removed also to remove both ovaries. In about fifty per cent of the cases we will have some signs of climacterium, that is: vasomotor disturbances. But it is an established fact that the artificial menopause where complete ablation has been done will last only a few months and the symptoms are much milder than during the natural climacterium.

Formerly I was too confident of getting my patient well right after the operation. I have found out, that I have made a great mistake in promising such a thing. In the first place it takes several months before the whole organism comes in proper relationship, and, secondly, it is of more importance not to state to the patient that both ovaries have been removed. The mental influence may work havoc with the brain cells and we certainly will get the blame. Statistics have shown that there is a great difference when the uterus has been removed and the ovaries left, and when the ovaries have been removed and the uterus left. Therefore, if the ovaries are removed, the uterus also should be removed. If we would look up those cases in which the psychoneuroses are present after abdominal extirpation of both ovaries, we would find that those in which the uterus has been removed at the same time do not present those symptoms complex and that only those are nervous wrecks where the uterus was not removed at the same time.

In conclusion I wish to state:

1. In all cases of infection of the adnexa, try and work conservatively.

2. Always extirpate a part of the wall of the uterus at the uterine end of the Fallopian tube.

3. If the ovaries are damaged to such an extent that same must be extirpated, always remove the uterus also.

4. The artifical menopause will produce vasomotor disturbances in only about fifty per cent of the cases and those symptoms are transient and will last only a few months.

5. Internal administration of corpus luteum extract will be of benefit only in those cases in which the uterus has been preserved and if the medical administration of corpus luteum extract does not improve the condition, hysterectomy should be done.

6. In specific infection of the adnexa, both tubes should be removed, even if the gross pathology is not very much pronounced.

7. If both ovaries are removed, this fact should not be presented direct to the patient to avoid psychic impressions, which would develop into psychoneurosis.

